# Financial subsidies, tax incentives, and new energy vehicle enterprises’ innovation efficiency: Evidence from Chinese listed enterprises

**DOI:** 10.1371/journal.pone.0293117

**Published:** 2023-10-25

**Authors:** Binhua Qian

**Affiliations:** School of Economy and Management, Zhejiang Business Technology Institute, Ningbo, China; Sichuan University, CHINA

## Abstract

Financial subsidies and tax incentives play essential roles in the innovation efficiency of enterprises. This paper selects Chinese listed NEV enterprises from 2010 to 2022 as a research sample and investigates various impacts under various circumstances. We find that both financial subsidies and tax incentives can promote the innovation efficiency of NEV enterprises. Compared to financial subsidies, tax incentives are more effective; the interaction between financial subsidies and tax incentives has a weaker impact on the innovation efficiency of NEV enterprises. Both financial subsidies and tax incentives have more potent innovation effects on enterprises with higher financing constraints. In addition, financial subsidies and tax incentives have a stronger innovation efficiency effect on private enterprises than state-owned enterprises. Further research shows that marketization and market distortion affect the innovation efficiency of NEV enterprises. This study provides a new perspective for understanding the effects of financial subsidies, tax incentives, and innovation efficiency of NEV enterprises, and the conclusions and suggestions are relevant references for the government to improve the quality of policy-making.

## 1 Introduction

Nowadays, China has become the world’s largest vehicle producer and seller. With the acceleration of China’s energy structure transformation, traditional fuel vehicles are gradually fading out of the market, while new energy vehicles(NEVs) have become the main development direction. Due to NEVs’ low pollution and emission, China has continuously introduced policies to promote the development of the NEV industry, in order to reduce pollution and emissions, and gain economic effect.

Since 2009, the Chinese government has focused on NEV development to achieve better promotion in the auto industry, and the government has issued a series of financial subsidy and tax incentive policies to promote the development of NEVs. As to financial subsidies for NEVs, they mainly carried out from both the demand and the supply sides. On the supply side, the government provides operational and innovation subsidies to NEV manufacturers. On the demand side, the government adopts centralized procurement and provides subsidies to consumers to reduce the purchasing fees. Regarding tax incentives for NEV enterprises, they cover both producers and consumers, and the whole process of R&D, production, sale, and use. There are two primary forms of tax incentives: tax exemptions and preferential tax rates. For example, the consumption tax exemption for lithium batteries, the consumption tax exclusion of pure electric vehicles and fuel cell passenger vehicles; the implementation of a low enterprise income tax rate of 15% for high-tech enterprises, R&D expenses deduction at 100%; and the exemption of vehicle tax and vehicle purchase tax for qualified NEV enterprises.

**Fig 1:**
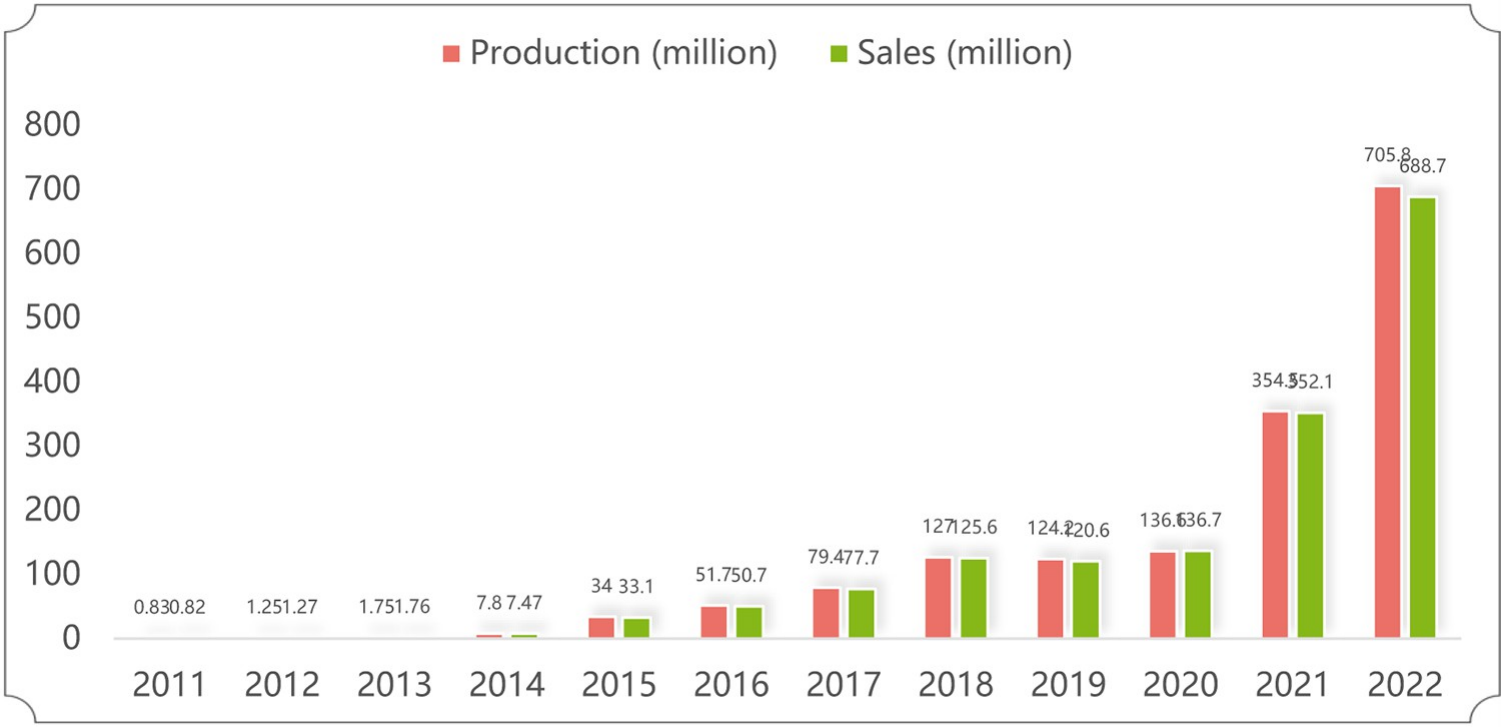
2011–2022 China’s NEV production and sales. (Source: Based on the data published by the Chinese Automobile Industry Association).

Under the implementation of financial subsidies and tax incentives, in recent eight years, China has achieved yearly growth in the production and sales of NEVs and has remained the world’s No. 1 production and sales volume(see Fig 1).

As ex-ante and ex-post policies, financial subsidies and tax incentives play pivotal roles in the innovation efficiency of NEV enterprises. For example, financial subsidies can provide NEV enterprises with sufficient cash flow [1], alleviate their financial difficulties and contribute to R&D investment more quickly. However, due to their threshold restrictions and funding amount, financial subsidies may discourage enterprises’ R&D enthusiasm [2]. In addition, the effects of financial subsidies and tax incentives on R&D innovation are also affected by geographical characteristics, enterprise scale, property rights, and other factors [3–5].

Recently, the effects of financial subsidies and tax incentives on innovation efficiency have become a debated topic, and literature has increasingly emerged and offered contrary conclusions. Some studies propose that financial subsidies and tax incentives positively impact on innovation efficiency. For example, they raise the innovation enthusiasm of enterprises [6–8]. Also, they can effectively reduce the positive externalities, high risks, and high costs of R&D activities, enable enterprises to focus on innovation efficiency [9–13]. Some studies propose that financial subsidies and tax incentives would crowde out enterprise innovation. Large enterprises would be more likely to increase innovation investment with financial subsidies and tax incentives received [14–17]. Besides, some studies compare financial subsidies and tax incentives, and draw contrary conclusions. Some studies propose that the effect of financial subsidies is superior to that of tax incentives [18, 19]., while some studies propose a non-linear relationship between financial subsidies, tax incentives, and enterprise innovations [20–23].

Till now, few studies have included financial subsidies, tax incentives, and innovation efficiency in a unified research framework, and there has not reached a consistent conclusion about the relationships between them. Some representative studies propose that only financial subsidies positively stimulated innovation performance because the "strong monitoring" feature of financial subsidies could alleviate opportunism in enterprises.

As a summary of the existing studies, they most focus on the relationship between two objects. In comparison, the relationship between these three objects has yet to reach a consistent conclusion, and fewer studies have included these objects in a unified research framework to investigate their intrinsic mechanisms, logical relationships and paths of action. Besides, several problems remain, such as: What are the roles of financial subsidies and tax incentives in the innovation efficiency of NEV enterprises? What are the role differences of financial subsidies and tax incentives for NEV enterprises with different financing constraints and ownership types?

Compared to the existing literature, our study makes several contributions to the literature. First, we combine financial subsidies, tax incentives, and innovation efficiency of NEV enterprises into a unified research framework. Second, our study includes the whole NEV manufacturers, including upstream and downstream enterprises in the industrial chain, so as to reflect the whole vehicle chain and policy effects. Third, to fully reflect the innovation efficiency of NEV enterprises, we calculate the innovation efficiency from the expected number of intellectual property authorizations and the loss of intellectual property output from unexpected outputs.

The remainder of this paper is assigned as follows. Section 2 provides theoretical analysis and research hypotheses. Section 3 describes the research design and data source. Section 4 analyzes the empirical results, including baseline regression, hysteresis effect, heterogeneity test, endogeneity test, financial constraint, and ownership heterogeneity analysis. We also conduct further research on marketization and market factor distortions. Section 5 gives conclusions and suggestions.

## 2 Theoretical analysis and research hypothesis

In this section, we theoretically analyze and compare the relationship among financial subsidies, tax incentives, and innovation efficiency.

### 2.1 Financial subsidies, tax incentives, and enterprise innovation efficiency

Scholars have different views on the relationship between financial subsidies, tax incentives, and innovation efficiency, and there exist two main viewpoints.

(1) Both financial subsidies and tax incentives have enhancing effects on enterprise innovation. Keizer et al. (2002) conducted telephone interviews with managers of small and medium-sized metal and electronics enterprises in the Netherlands. They revealed the vital role of financial subsidies on enterprise innovation performance [24]. González et al. (2005) analyzed the role of financial subsidies on enterprise innovation performance with a sample of 2000 Spanish manufacturing enterprises, and the results showed that financial subsidies stimulated enterprise innovation and no private funds were squeezed out [25]. Gerarden (2022) examined the role of financial subsidies on solar panel manufacturers and found that financial subsidies led to more innovations in international enterprises, resulting in lower prices and more market adoption [10]. Wang et al. (2022) proved that in the case of higher buyer power, government subsidies would significantly promote the R&D investment of enterprises and the positive effect was not affected by nature of the enterprise’s ownership [26].

(2) Financial subsidies and tax incentives crowd out enterprise innovation. Wu et al. (2022) used financial data of Chinese listed companies from 2007–2017 to explore the impact of financial subsidies on innovation investment of new energy enterprises. They found that the size of subsidies had an inverted U-shaped relationship with enterprises’ innovation investment. The higher subsidies were, the more crowding-out severe effect on enterprises’ R&D investment existed [14]. Boeing (2022) finished research with panel data of Chinese provinces over the period 2000–2010 and found that public R&D subsidies allocated to medium and large enterprises increased total R&D investment, but reduced privately financed R&D investment, which means there was a partial crowding-out effect of financial subsidies on enterprise innovation [15]. Yang (2017) pointed out that after the implementation of financial policy, R&D manipulation phenomenons would appear, which was contrary to the original purpose of financial policies and also led to a decline in innovation performance [27].

In the initial stage, most of NEV enterprises will inevitably face some constraints, such as small scale, low capital, and backward technology. At this time, financial subsidies will help them to invest more in R&D, thus reducing the technical threshold and lowering the cost of technological innovation of enterprises, as well as improving the technological innovation enthusiasm. As a result, financial subsidies can stimulate NEV enterprises to create higher innovation returns for advanced technologies.

Therefore, this paper proposes hypothesis 1:

H1: Financial subsidies positively affect the innovation efficiency of NEV enterprises.

Scholars have found that R&D investments positively correlate with the tax incentives. The more tax incentives are, the more R&D investment increases. In general, tax incentives can positively affect technological innovation in NEV enterprises by reducing the marginal cost of innovation because they can alleviate the financing constraints and enhance the innovation risk compensation. On one hand, R&D innovation needs capital support, while tax incentives can provide sufficient profit to reduce expenditures. On the other hand, the tax incentives are conducive for enterprises to enhance their innovation enthusiasm and, to a certain extent, reduce the uncertainty caused by innovation activities. In addition, the potential adverse effects of the NEV enterprises are compensated by the tax incentives to some extent.

So, this paper proposes hypothesis 2:
H2: Tax incentives positively affect the innovation efficiency of NEV enterprises.

### 2.2 Interaction analysis of the role of financial subsidies and tax incentives on innovation

As the research progresses, scholars focus on the interaction analysis of financial subsidies and tax incentives, and conduct many in-depth analyses. The main conclusions can be summarized into the following categories.

(1) Financial subsidies and tax incentives positively affect innovations. Le (2016) studied the effect of R&D financial subsidies on the innovation output of New Zealand enterprises, and the results showed that financial subsidies promoted innovations in both large and small enterprises with less than 50 employees, and the promotion effects had no significant differences [28].

(2) Financial subsidies’ incentive effect differs from that of tax incentives. Tong et al. (2023) found that financial subsidies stimulated technological innovation in NEV enterprises, and tax incentives had no significant effects [18]. Busom et al. (2014) found that financial subsidies were more effective in encouraging enterprises to undertake R&D innovations [19].

(3) There is a non-linear relationship among financial subsidies, tax incentives, and enterprise innovations. Qin (2022) found that the innovation promotion effects of financial subsidies tend to increase at the initial stage, then decrease at the developing stage, and were more likely to lead to low-quality innovations [20].

In our point of view, financial subsidies and tax incentives have consistent goals. They both aim to improve the R&D and innovation capabilities of enterprises. For example, financial subsidies increase enterprises’ R&D investment from a "individual" perspective, while tax incentives exert efforts to increase enterprises’ R&D investment from a "general" perspective. The government can leverage the advantages of these policies and, through a "individual-general combination" regulatory approach, guides enterprises to invest more in R&D fields encouraged by the government, thereby enhancing the resilience of economic and social development. Financial subsidies are "pre-incentive", which act before research and development investment; tax incentives are "post-incentive", which act after R&D investment. Financial subsidies are rapid and direct, but relatively short; while tax incentives are relatively long. Table 1 illustrates the interaction of them. If these policies are combined, they can cover the entire process of enterprise R&D activities. Their synergies can form a "1+1>2" pattern and positively impact enterprise R&D investments.

**Table 1:** Interaction of financial subsidies and tax incentives on R&D.

	Financial subsidies	Tax Incentives
Stage	Before R&D investment	After R&D investment
Scope	Government-selected R&D areas and projects	All R&D activities
Recipients	Governments select funding priorities and projects	Enterprises choose R & D projects independently
Fairness	Differentiated, may distort equity	Undifferentiated and fair
Speed of action	Quick, direct and obvious	Weak at the beginning, better in the long term
Implementation Costs	Low	High

To this end, this paper proposes hypothesis 3:

H3: The interaction of financial subsidies and tax incentives positively affects the innovation efficiency of NEV enterprises.

**Fig 2:**
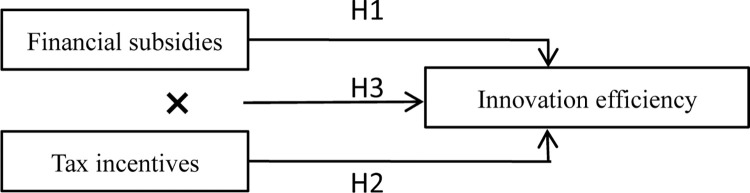
Theoretical model.

Fig 2 illustrates the theoretical model.

## 3 Research design

### 3.1 Sample and data collection

In this paper, we collect 603 listed NEV enterprises of China stockmarket from 2010–2022, and match the financial data in the CSMAR database (http://www.gtarsc.com/). The main reasons are as follows: First, compared with small and medium-sized enterprises, the financial data of listed enterprises are more transparent and have higher credibility. Second, China has promoted the NEV development since 2009, and data from 2010 to the present could better reflect the innovation effect of financial subsidies and tax incentives in the long run. Finally, we consider the entire vehicle production of and include upstream and downstream enterprises in the NEV industry chain to reflect the effect as much as possible.

We have taken the following preprocessing measures for the data:(1) To avoid the influence of some enterprises’ liabilities over their assets, ST and *ST enterprises are excluded. (2) To avoid some loss-making enterprises from affecting the empirical results, enterprises whose effective tax rate or total profit is less than zero are excluded. (3)To avoid data distortion, those data that do not comply with accounting principles are deleted, which include enterprises with current assets greater than total assets, enterprises with profits more significant than operating income, enterprises with zero total assets, and enterprises with abnormal financial subsidies. (4)To avoid the effect of outliers on the regression results, a two-sided winsorize tailing process is applied to all data at the 1% level. These enterprises include vehicle manufacturers, spare parts, and related equipment manufacturing enterprises. The obtained data are unbalanced panel data. Stata 16.0 is employed to calculate these data.

### 3.2 Variables

#### 3.2.1 Explained variables: The innovation efficiency of NEV enterprises

Most previous studies have carefully measured the innovation efficiency of enterprises based on R&D output, such as the number of patent applications and the number of patents granted. It is difficult to measure the total innovation efficiency, because only the R&D output is considered, while the R&D input is omitted. Based on previous studies, this paper uses the data envelopment analysis (DEA) method to calcute the innovation efficiency. It considers the super efficiency of the non-expected output (SBM-Undesirable) model to comprehensively measure the innovation efficiency of NEV enterprises. The specific formulas are as follows.


ρ*=min 1−1N∑n=1NSnxXk,nt1+1M+1(∑m=1MSmyyk,mt+∑l=1LSibbk,tt)s.t.{∑k=1,k≠jKλktXk,nt+snx=Xk,nt,n=1,…,N∑k=1,k≠jKλktXk,nt−smy=yk,mt,m=1,…,M∑k=1,k≠jKλktXk,tt+Stb=bk,it,i=1,…,Iλkt≥0,snx≥0,smy≥0,Sib≥0,k=1,…,K

Based on the DEA model of non-expected output, this paper uses the total R&D expenditure and the total number of technicians to represent the capital and labor inputs, and the number of patents granted to define the output. In the meantime, the difference between the number of patent applications and patents granted by the enterprise is selected to represent the unavoidable innovation loss.

#### 3.2.2 Explanatory variables: Financial subsidies and tax incentives

In this paper, the explanatory variables are financial subsidies and tax incentives.

Financial subsidies (Subsidy): Take the number of financial subsidies obtained by the enterprises in the non-operating income section of the annual reports and take the data value logarithmically.

Tax incentives (Tax): Take the value of (nominal tax rate—effective tax rate) × total profit to illustrate the tax incentives received by the NEV enterprises. Here, the nominal tax rate refers to the tax rate specified in the tax law, while the effective tax rate refers to the actual tax rate borne. The higher the value, the more tax incentives the enterprise enjoys. Also, we take the data value logarithmically.

#### 3.2.3 Control variables

According to the study of Hellmann (2011) [29] and Xie (2022) [12], we choose the following as control variables: return on net assets, age of business establishment, shareholder shareholding ratio, labor intensity, enterprise size, gearing ratio, fixed assets ratio, Tobin’s Q value and level of cash holdings.

1. Return on net assets (Roe): the net profit divided by total asset data.
2. Age of business establishment (Age): current year—establishment year+1.
3. Shareholder shareholding ratio (Share): the shareholding ratio of the enterprise’s largest shareholder.
4. Labor intensity (Labor): the logarithm of total employee number.
5. Enterprise size (Size): the logarithm of the total assets.
6. Gearing ratio (Tdr): the total enterprise liabilities divided by total assets.
7. Fixed assets ratio (Far): the net fixed assets divided by total assets.
8. Tobin’s Q value: enterprise value divided by total assets.
9. Level of cash holdings (Cash): the logarithm of the closing balance of cash and cash equivalents.

Table 2 shows the details of these variables.

**Table 2:** Description of variables.

Types	Variables	Symbols	Definitions
Explained variable	Innovation efficiency	RD	calculated by using the DEA model
Explanatory variables	Financial subsidies	Subsidy	ln(financial subsidies)
	Tax incentives	Tax	ln((nominal tax rate—effective tax rate) × total profit)
Control variables	Return on net assets	Roe	net profits/total assets
	Age of business establishment	Age	current year—establishment year+1
	Shareholder shareholding ratio	Share	the shareholding ratio of the enterprise’s largest shareholder
	Labor Intensity	Labor	ln(total number of employees)
	Enterprise size	Size	ln(total assets)
	Gearing ratio	Tdr	total liabilities / total assets
	Fixed assets ratio	Far	net fixed assets/total assets
	Tobin’s Q value	TobinQ	enterprise value / total assets
	Level of cash holdings	Cash	ln (closing balance of cash and cash equivalents)

### 3.3 Models

Refering to Wang (2022) [26] and Shi (2023) [16], this paper constructs the basic regression models to test hypotheses 1–3, and fixed-effects model is employed. A regression analysis with the stepwise addition of core explanatory variables is adopted to compare the different effects that differentiated policies may induce.

$$RD_{it}=\alpha +\beta_{1}\mathrm{Subsid}y_{i,t}+\beta_{2}X_{i,t}+\gamma \eta_{t}+\lambda \eta_{i}+\varepsilon_{i,t}$$ (1)

$$RD_{it}=\alpha +\beta_{1}{\mathrm{Tax}}_{i,t}+\beta_{2}X_{i,t}+\gamma \eta_{t}+\lambda \eta_{i}+\varepsilon_{i,t}$$ (2)

$$RD_{it}=\alpha +\beta_{1}{\mathrm{Subsidy}}_{i,t}+\beta_{2}{\mathrm{Tax}}_{i,t}+\beta_{3}X_{i,t}+\gamma \eta_{t}+\lambda \eta_{i}+\varepsilon_{i,t}$$ (3)

Where RD is the innovation efficiency of NEV enterprises, i and t represent enterprise and year respectively, α is a constant term, Subsidy_i,t_ is the financial subsidies received by the ith enterprise in the year t, Tax_i,t_ is the tax incentives received by enterprises, X_i,t_ is the control variable, η_t_ represents the time fixed effect, η_i_ represents the individual fixed effect, and ε_i,t_ represents the random disturbance term.

The effects of financial subsidies and tax incentives on innovation efficiency are interactive. Therefore, based on the original model, the following interaction effect model is constructed by adding the interaction term.


$$RD_{it}=\beta_{1}Subsidy_{i,t}+\beta_{2}Tax_{i,t}+\beta_{3}Subsidy_{i,t}\times Tax_{i,t}+\beta_{4}X_{i,t}+\gamma \eta_{t}+\gamma \eta_{i}+\varepsilon_{i,t}$$ (4)

## 4. Results and analysis

### 4.1 Descriptive statistics

Table 3 reports the descriptive statistics of all variables. The deviations of cash holdings, gearing ratio, tax incentives, and age of establishment of NEV enterprises are large. Regarding innovation efficiency, they vary greatly. The smallest is zero, while the largest is 2.514. Regarding labor intensity, they do not vary much. In terms of cash holdings, the enterprises with the most minor cash holdings are negative. In terms of the gearing ratio, the deviation between the most significant and most minor gearing ratios is about ten times. In terms of tax incentives, some enterprises do not enjoy tax incentives, and there is a large difference between them. In terms of the age of enterprises, they vary between 13 and 28 years, and there is a significant difference among them. The lowest value of innovation efficiency is 0, while the dispersion of innovation efficiency among them is not high. In addition, the financial subsidies enjoyed by the NEV enterprises are similar.

**Table 3:** Descriptive statistical results.

Varname	Obs	Mean	Std. Dev	Min	Max
RD	6162	0.221	1.298	0.000	2.514
Subsidy	6162	13.903	1.623	8.819	15.265
Tax	6162	12.375	3.943	0.000	18.651
Roe	6162	0.059	0.087	-0.127	0.243
Age	6162	15.365	4.853	13.000	28.000
Share	6162	0.215	0.145	0.136	0.416
Labor	6162	7.056	1.106	5.928	9.784
Size	6162	20.319	1.278	17.173	21.821
Tdr	6162	2.853	3.943	1.046	11.167
Far	6162	0.435	0.213	0.123	0.736
TobinQ	6162	1.184	1.865	0.755	5.144
Cash	6162	5.379	7.293	-2.318	15.185

### 4.2 Main results

In this section, we conduct a series of empirical analyses about the impact of financial subsidies and tax incentives on NEV enterprises’ innovation, including baseline regression, heterogeneity tests, endogeneity tests, and robustness tests.

Referring to Xie (2022) [12], before empirical analyses, we take a series of test. Mixed OLS regression tests reveal the VIF across all the models, which is far less than the suggested threshold of 10 for risk of multicollinearity, suggesting that multicollinearity is not a substantial issue on the variables. Considering the influence of time change on the regression results, we adopted the panel data model method (i.e. the Hausman test). The Hausman test result shows that each model’s Prob > chi2 value is less than 0.05, demonstrating that the null hypothesis is rejected. So this paper excludes random-effects models and selects fixed effects models. Concerning the determination of time fixed or individual fixed, this paper selects the individual fixed-effects model and passed the F-test.

#### 4.2.1 Baseline regression

Table 4 reports the baseline regression results. The results in columns (1) to (5) show that financial subsidies and tax incentives are significantly positive during policy implementation. In contrast, the regression coefficients of financial subsidies are smaller than those of tax incentives, and the innovation efficiency effect of financial subsidies is weaker than that of tax incentives. Specifically, the results in column (1) show that if the financial subsidies increase by 1 unit, the enterprises’ innovation efficiency will increase by 0.0361 units on average; the results in column (2) show that if the tax incentives increase by 1 unit, the enterprises’ innovation efficiency will increase by 0.0381 units on average. After adding enterprise-level control variables, financial subsidies and tax incentives still significantly affect innovation efficiency.

**Table 4:** Baseline regression results.

Variables	(1)	(2)	(3)	(4)	(5)
	RD	RD	RD	RD	RD
Subsidy	0.0361** (0.015)		0.0336** (0.016)		0.0332** (0.017)
Tax		0.0381*** (0.011)		0.0443*** (0.018)	0.0446*** (0.017)
Size			0.002 (0.002)	0.005* (0.003)	0.000 (0.002)
Roe			0.015** (0.007)	0.011** (0.005)	0.031** (0.015)
Cash			0.006*** (0.002)	0.031*** (0.012)	0.017*** (0.005)
Share			-0.026** (0.011)	-0.019** (0.006)	-0.052** (0.022)
Tdr			0.002 (0.023)	0.004 (0.035)	0.003 (0.025)
Age			0.004*** (0.001)	0.009*** (0.004)	0.008*** (0.002)
Labor			0.008** (0.004)	0.007** (0.003)	0.004** (0.002)
Far			0.003 (0.023)	0.003 (0.035)	0.004 (0.013)
TobinQ			0.008*** (0.002)	0.006** (0.003)	0.001*** (0.001)
Constant	0.082***	0.056***	0.032***	0.027***	0.021*
	(0.005)	(0.011)	(0.004)	(0.003)	(0.011)
Observations	6162	6162	6162	6162	6162
R-squared	0.511	0.509	0.632	0.637	0.630
Industry fixed effects	yes	yes	yes	yes	yes
Time fixed effects	yes	yes	yes	yes	yes

Note: Clustering robust standard errors are in parentheses, clustering at the individual enterprise level.

*, ** and *** denote coefficients significant at the 10%, 5% and 1% levels, respectively

In terms of control variables, the coefficient of Roe is significantly positive, which indicates that an increase in profitability will lead to an increase in innovation efficiency. The coefficient of cash holding level(Cash) is positive with the innovation efficiency, probably because enterprises with sufficient cash are more courageous to increase R&D investment, thus enhancing innovation efficiency. The shareholder shareholding ratio (Share) is significantly negatively correlated with innovation efficiency, mainly because the more decentralized the shareholding distribution is, the less likely it is to decentralize the interests of shareholders. If R&D investment increases, it will lead to a decline in profits in the short term, so the enterprise is unwilling to spend more on R&D investment. The coefficient of the age of enterprise establishment (Age) is significantly positive, which indicates that the longer the enterprise exists, the stronger the factor concentration and risk resistance ability are. The coefficient of Labor is significantly positive, indicating that the larger the enterprise is, the it will be more willing to increase its R&D investmen, thus enhancing its market competitiveness. The coefficient of Tobin’s Q is significantly positive with the innovation efficiency of enterprises, indicating that the higher the market valuation of enterprises is, the higher the innovation efficiency is.

#### 4.2.2 The heterogeneity tests

In this subsection, we conduct heterogeneity tests from the financing constraints and the ownership of NEV enterprises.

*(1) Financing constraints*. The innovation capacity of enterprises and their willingness determine whether the financial subsidies and tax incentives can improve innovation efficiency, and the difference influences the policy effects in the level of financing constraints inherent. Acharya and Xu (2013) argued that financing constraints profoundly impacted investment decisions and business activities, thus significantly increasing their cash flow sensitivity, which became an important reason to hinder technological progress [30]. Fazzari et al. (1988) pointed out that when capital market conditions were imperfect, enterprises’ external financing costs would increase [31]. Song et al. (2011) pointed out that when China was in the transition stage, many non-market factors, such as the relationship with the government and financing channels would prevented enterprises from obtaining external financing at a lower cost [32]. Solving the capital demand problem for enterprises could enable them invest more in R&D activities and increase innovation outputs. Specifically, when it was relatively complex for enterprises to obtain funds, financial subsidies and tax incentives could effectively reduce the R&D difficulties. For example, tax incentives had a strong marginal incentive effect to effectively alleviate the financing constraints, stimulate them to put resources into new technology fields, and promote innovation.

Concerning Hadlock and Pierce (2009) [33, 34], the SA index of NEV enterprises is constructed, and the smaller the SA index is, the more severe the financing constraint is. This paper takes the absolute value of SA. It groups the SA values according to the median, where a value greater than the median is 1, indicating a more vital financing constraint, and the opposite is 0, indicating a weaker financing constraint.


$$\mathrm{SA}=‐0.737\times \mathrm{size}+0.043\times {\mathrm{size}}^{2}‐0.040\times \mathrm{age}$$ (5)

According to the above analysis, the innovation efficiency effects of financial subsidies and tax incentives differ in the financing constraint situations. Table 5 reports the heterogeneity analysis of financing constraints. The coefficient of financial subsidies is 0.0389 when the financing constraint is high and 0.0279 when the financing constraint is low. The coefficient of financial subsidies in the group with higher financing constraints is slightly higher than that of the group with lower financing constraints, indicating that when the financing constraints are higher, the impact of financial subsidies on the innovation efficiency of NEV enterprises is also more substantial. As for tax incentives, the coefficient of tax incentives on the innovation efficiency of enterprises is 0.0462 when the market financing constraint is high, which is greater than that of a low financing constraint. Overall, it can be seen that the higher the financing constraint is, the higher the coefficient of tax incentives is. When other conditions remain unchanged, financial subsidies and tax incentives have stronger impacts on the innovation efficiency of NEV enterprises.

**Table 5:** Heterogeneity analysis of financing constraints.

	(1)	(2)	(3)	(4)	(5)	(6)
	Full sample	Low financing constraints	High financing constraints	Full Sample	Low financing constraints	High financing constraints
VARIABLES	RD	RD	RD	RD	RD	RD
Subsidy	0.0356** (0.017))	0.0279* (0.015))	0.0389** (0.002)			
Tax				0.0436*** (0.018))	0.0040** (0.002))	0.0462*** (0.009)
Size	0.0023 (0.002)	0.0011 (0.001))	0.0018 (0.002))	0.0053* (0.003))	0.0023 (0.019))	0.0052 (0.019)
Roe	0.0151** (0.007)	0.0176** (0.008)	0.0190** (0.009)	0.0101** (0.005)	0.0179** (0.007)	0.0159** (0.007)
Cash	0.0058*** (0.002)	0.0519* (0.027)	0.0534* (0.027)	0.0311*** (0.012)	0.0311** (0.013)	0.0355*** (0.011)
Share	-0.0276** (0.011)	-0.0414*** (0.013)	-0.0261*** (0.009)	-0.0139** (0.006)	-0.0168** (0.008)	-0.0181** (0.007)
Tdr	0.0012 (0.023)	0.0018 (0.002)	0.0011 (0.003)	0.0045 (0.035)	0.0041* (0.002)	0.0045 (0.011)
Age	0.0041*** (0.001)	0.0368*** (0.006)	0.0386*** (0.005)	0.0095 *** (0.004)	0.0094*** (0.003)	0.0098*** (0.002)
Labor	0.008** (0.004)	0.0083*** (0.002)	0.0087*** (0.002)	0.0062** (0.003)	0.0064*** (0.002)	0.0061** (0.003)
Ta	0.003 (0.023)	0.0031 (0.002)	0.0039* (0.002)	0.0026 (0.035)	0.0023 (0.004)	0.0029 (0.002)
TobinQ	0.008*** (0.002)	0.0085** (0.004)	0.0078*** (0.002)	0.0061** (0.003)	0.0059** (0.003)	0.0056*** (0.001)
Constant	0.0322*** (0.004)	0.0269*** (0.005)	0.0306*** (0.004)	0.0263*** (0.003)	0.0257*** (0.005)	0.0259*** (0.005)
Observations	6162	2464	3698	6162	2464	3698
R-squared	0.629	0.656	0.638	0.635	0.667	0.666
Industry fixed effects	yes	yes	yes	yes	yes	yes
Time fixed effects	yes	yes	yes	yes	yes	yes

Note: Clustering robust standard errors are in parentheses, clustering at the individual firm level.

*, ** and *** denote coefficients significant at the 10%, 5% and 1% levels, respectively

*(2) Enterprise ownership heterogeneity analysis*. Due to the difference in enterprise ownership, state-owned enterprises(SOEs) and private enterprises differ in obtaining financial subsidies and tax incentives. Theoretically, SOEs would use the obtained financial and tax resources to enhance innovation efficiency, so as to improve innovation efficiency. However, research has revealed that SOEs must pay more attention to the government’s resource inclination. The reasons are: on the one hand, SOEs are supervised and operated by the government, they have certain advantages over private enterprises in resource endowment, and their operating profits need to be paid to the government. Therefore, when SOEs face insufficient profits or financial loss, the government will probably provide tax incentives and financial subsidies to them. On the other hand, SOEs can obtain more resources than private enterprises under their close ties with the government. The resource advantage will make them avoid risky and innovative inputs and maintain competitive advantages in the market competition [35–38].

In this paper, enterprise ownership is set as a dummy variable, and the innovation efficiency effects on different ownerships are compared by dividing the selected sample into two parts: one part of SOEs and the other part of private enterprises. Table 6 reports the results. Compared to SOEs, financial subsidies and tax incentives have a more substantial effect on the innovation efficiency of private enterprises, mainly because SOEs tend to be in an advantageous position in market competition and enjoy government policies, while private enterprises are in a more severe competitive market situation. Private enterprises have to be more effective to gain and use subsidies. Therefore, financial subsidies and tax incentives are more prominent in enhancing the innovation efficiency of private enterprises.

**Table 6:** Heterogeneity analysis of enterprise ownership.

	(1)	(2)	(3)	(4)	(5)	(6)
	Full sample	State-owned	Private	Full sample	State-owned	Private
VARIABLES	RD	RD	RD	RD	RD	RD
Subsidy	0.0356** (0.017)	0.02216** (0.009)	0.0374*** (0.003)			
Tax				0.0436*** (0.018)	0.0135*** (0.003)	0.0325*** (0.002)
Size	0.0023 (0.002)	0.0032 (0.011)	-0.0052 (0.011)	0.0053* (0.003)	0.0025 (0.002)	0.0021 (0.003)
Roe	0.01516** (0.007)	0.01596** (0.007)	0.01796** (0.008)	0.01016** (0.005)	0.0119*** (0.001)	0.0151*** (0.002)
Cash	0.0058*** (0.002)	0.0044* (0.002)	0.0048*** (0.001)	0.0311*** (0.012)	0.0271*** (0.009)	0.0323*** (0.002)
Share	-0.02766** (0.011)	-0.0231*** (0.003)	-0.0328*** (0.002)	-0.01396** (0.006)	-0.0187*** (0.007)	-0.01666** (0.007)
Tdr	0.0012 (0.023)	0.0015 (0.002)	0.0019 (0.003)	0.0045 (0.035)	0.0039* (0.002)	0.0031 (0.002)
Age	0.0041*** (0.001)	0.0021* (0.002)	0.0036*** (0.001)	0.0095*** (0.004)	0.0083*** (0.002)	0.0086*** (0.002)
Labor	0.0086** (0.004)	0.00616** (0.003)	0.0063*** (0.002)	0.00626** (0.003)	0.0046* (0.002)	0.0048*** (0.001)
Far	0.003 (0.023)	-0.0007 (0.002)	0.0031 (0.002)	0.0026 (0.035)	0.0032 (0.025)	0.0048 (0.025)
TobinQ	0.008*** (0.002)	0.0166*** (0.003)	0.0075*** (0.001)	0.00616** (0.003)	0.00516** (0.002)	0.00536** (0.003)
Constant	0.0322*** (0.004)	0.0215*** (0.002)	0.0459*** (0.005)	0.0263*** (0.003)	0.0218*** (0.003)	0.0379*** (0.002)
Observations	6162	2730	3432	6162	2730	3432
R-squared	0.629	0.578	0.628	0.635	0.679	0.632
Industry fixed effects	yes	yes	yes	yes	yes	yes
Time fixed effects	yes	yes	yes	yes	yes	yes

Note: Clustering robust standard errors are in parentheses, clustering at the individual firm level.

*, ** and *** denote coefficients significant at the 10%, 5% and 1% levels, respectively

### 4.3 Endogeneity tests

The endogeneity of this paper mainly comes from two aspects. On one hand, there exist causal relationships between each other. Enterprises with solid innovation efficiency will send positive signals to the outside world, which will make them more easier to obtaine financial and tax supports. On the other hand, although we control those essential characteristics of NEV enterprises in the regression model, some variables may need to be added to affect the innovation efficiency. The above situations may lead to endogeneity issues, resulting in biased estimation results. So, this paper adopts a two-stage least squares method to solve endogeneity.

Referring to Yao et al. (2011) [39], this paper uses financial subsidies and tax incentives with a lag of one period as instrumental variables (IV) to control endogeneity. Table 7 reports the regression results. Column(1) shows the regression results of industry-level clustering, while columns(2)-(5) show the impacts of financial subsidies and tax incentives with a lag of one period on the innovation efficiency of NEV enterprises. In column(2) and column(4), the regression coefficient of L.Subsidy is significant at the 10% level and 5% level separately. In column(2) and column(3), the regression coefficient of L.Tax is significant at the 5% level. The results indicate that after the endogeneity issue is resolved, financial subsidies and tax incentives have positively affected the innovation efficiency of NEV enterprises. The regression results of other control variables are also consistent with the desired effect.

**Table 7:** The endogeneity test(IV) results.

	(1)	(2)	(3)	(4)	(5)
VARIABLES	RD	RD	RD	RD	RD
Subsidy	0.034** (0.015)				
Tax	0.041*** (0.008)				
L.Subsidy		0.002* (0.001)		0.002** (0.011)	
L.Tax		0.011** (0.005)	0.083** (0.042)		
Size	0.003* (0.002)	0.006 (0.043)	0.007 (0.091)	0.004 (0.005)	0.002 (0.018)
Roe	0.062** (0.029)	0.058** (0.024)	0.018** (0.013)	0.016** (0.007)	0.031*** (0.009)
Cash	0.002** (0.001)	0.008*** (0.002)	0.001** (0.005)	0.002** (0.001)	0.004** (0.002)
Share	-0.014 (0.022)	-0.006 (0.023)	-0.062** (0.031)	-0.027** (0.011)	-0.001 (0.002)
Tdr	0.001 (0.043)	0.009 (0.026)	0.007 (0.093)	0.002 (0.005)	0.003 (0.068)
Age	0.072*** (0.012)	0.0089** (0.002)	0.093* (0.053)	0.005 (0.039)	0.049** (0.025)
Labor	0.008*** (0.002)	0.023*** (0.015)	0.004*** (0.001)	0.007** (0.003)	0.008*** (0.002)
Far	0.005 (0.043)	0.004 (0.039)	0.005 (0.064)	0.006 (0.063)	0.007 (0.044)
TobinQ	0.007*** (0.002)	0.006*** (0.002)	0.009*** (0.003)	0.015* (0.009)	0.007 (0.023)
Constant	0.086*** (0.002)	0.006*** (0.002)	0.009*** (0.003)	0.015* (0.009)	0.007 (0.023)
Observations	6162	6162	6162	6162	6162
R-squared	0.663	0.585	0.598	0.585	0.638
Industry fixed effects	yes	yes	yes	yes	yes
Time fixed effects	yes	yes	yes	yes	yes

Note: Clustering robust standard errors are in parentheses, clustering at the individual firm level.

*, ** and *** denote coefficients significant at the 10%, 5% and 1% levels, respectively

### 4.4 Robustness tests

Robustness checks are conducted by replacing core variables for financial subsidies and tax incentives.

Concerning Wang et al. (2022) [26], the replacement of the financial subsidy is calculated by the formula below:

Subsidy1 = financial subsidy/net profit.

Concerning Li (2023) [40], the replacement of the tax incentive is calculated by the formula below:

Tax1 = all refund taxes received / (all refund taxes received + all taxes paid).

The estimation of innovation efficiency is calculated a second time, and the results are shown in Table 8. The explained variable is innovation efficiency (RD), and the explanatory variables are financial subsidies (subsidy, subsidy1) and tax incentives (tax, tax1), respectively. The control variables remain consistent with the regressions. The results show no significant changes in the core variables, indicating that the results of the benchmark regressions are robust.

**Table 8:** Robustness checks after replacing core variables.

	(1)	(2)	(3)
Variables	RD	RD	RD
Subsidy		0.052*** (0.021)	
Tax	0.041** (0.022)		
Subsidy1	0.022** (0.011)		0.047*** (0.015)
Tax1		0.086*** (0.029)	0.067** (0.028)
Size	0.013 (0.011)	0.005 (0.032)	0.004 (0.011)
Roe	0.064*** (0.026)	0.029** (0.012)	0.056** (0.025)
Cash	0.001 (0.002)	0.009*** (0.003)	0.008** (0.004)
Share	-0.039* (0.002)	-0.003 (0.011)	0.031** (0.015)
Tdr	0.004 (0.093)	0.008 (0.013)	0.003 (0.089)
Age	0.029* (0.018)	0.045*** (0.021)	0.056 (0.043)
Labor	0.009*** (0.001)	0.019*** (0.031)	0.009*** (0.001)
Far	0.009 (0.087)	0.004 (0.079)	0.005 (0.098)
TobinQ	0.005* (0.003)	0.009* (0.005)	0.007*** (0.001)
Constant	0.031*** (0.014)	0.077*** (0.023)	0.091* (0.053)
Observations	6162	6162	6162
R-squared	0.628	0.815	0.698
Industry fixed effects	yes	yes	yes
Time fixed effects	yes	yes	yes

Note: Clustering robust standard errors are in parentheses, clustering at the individual enterprise level.

*, ** and *** denote coefficients significant at the 10%, 5% and 1% levels, respectively

### 4.5 Further analysis

This subsection explores the mechanism of regional characteristics on the relationship of financial subsidies, tax incentives, and NEV innovation efficiency, including marketization and factor market distortion.

#### 4.5.1 The mechanism of marketization

In the process of transitioning to a market economy system, there exist specific regional marketization differences. Regions with higher marketization processes have relatively sound financial and property rights protection systems. In those regions, the market plays a decisive role in resource allocation, and better protects corresponding R&D achievements. On the contrary, in those regions with lower marketization processes, there will be "rent-seeking" phenomena when implementing the financial subsidy and tax incentive policies, which will lead to distorted public resource allocations.

This paper uses the marketization index of the China Provincial Marketization Index Report proposed by Wang et al. (2022) to indicate the degree of marketization. Table 9 shows the regression results. Column (1) includes financial subsidies and marketization levels, as well as the interaction between them; column (2) contains tax incentives, the level of marketization, and the interaction between them, while column (3) contains all variables. It can be seen that the interaction coefficients are significantly positive, and they pass statistical tests at the 5% level, which indicates that under the same incentive situation, the higher the marketization process in regions is, the stronger the incentive effects of financial subsidies and tax incentives on innovation efficiency of NEV enterprises are.

**Table 9:** The regression results of marketization.

	(1)	(2)	(3)
Variables	RD	RD	RD
Subsidy	0.029* (0.017)		0.009* (0.005)
Marketization	0.003** (0.001)	0.008** (0.041)	0.003** (0.016)
Subsidy×Marketization	0.067** (0.031)		0.052** (0.025)
Tax		0.045** (0.021)	0.098*** (0.093)
Tax×Marketization		0.071* (0.043)	0.077** (0.035)
Size	0.033* (0.018)	0.001 (0.001)	0.001 (0.011)
Roe	0.035*** (0.006)	0.058** (0.023)	0.012** (0.006)
Cash	0.048* (0.025)	0.030** (0.015)	0.091* (0.051)
Share	-0.022** (0.011)	-0.053* (0.028)	-0.001 (0.014)
Tdr	0.006* (0.003)	0.003 (0.089)	0.001 (0.018)
Age	0.067* (0.035)	0.032** (0.016)	0.069** (0.032)
Labor	0.016** (0.008)	0.003 (0.043)	0.006*** (0.003)
Far	-0.000 (0.000)	0.007 (0.053)	0.008 (0.032)
TobinQ	0.009*** (0.003)	0.003 (0.015)	0.034* (0.002)
Constant	0.079** (0.039)	0.053*** (0.018)	0.042* (0.023)
Observations	6162	6162	6162
R-squared	0.437	0.487	0.567
Region fixed effects	yes	yes	yes
Time fixed effects	yes	yes	yes

Note: Clustering robust standard errors are in parentheses, clustering at the individual enterprise level.

*, ** and *** denote coefficients significant at the 10%, 5% and 1% levels, respectively

#### 4.5.2 The mechanism of factor market distortion

In the process of market-oriented transformation, due to government intervention and regulation, there may exist factor market distortions. In regions with relatively high degrees of distortion, enterprises are more willing to invest resources to obtain excess profits and strengthen connections with local governments rather than invest resources in innovations with relatively high risks. Similarly, enterprises that have achieved excess returns in the allocation of production factors will use such subsidies to strengthen their relationships with local governments. Meanwhile, the market will affect the distribution of financial subsidies [41, 42].

This paper uses the regional marketization and market distortion indexes to measure the degree of distortion in the regional factor market. These two indexes are also recorded in China Provincial Marketization Index Report proposed by Wang et al. (2022). Table 10 reports the regression results. Column (1) explores the impact of financial subsidies, factor market distortions, and their interaction on the innovation efficiency of NEV enterprises. It can be seen that financial subsidies have a positive, stimulating effect on innovation efficiency. In contrast, they have an inhibitory effect on the innovation efficiency of NEV enterprises under factor market distortions. Column (2) includes tax incentives, factor market distortions, and the interaction term. The results show that tax incentives positively impact innovation efficiency, while factor market distortion and the interaction term negatively impact innovation efficiency. Column (3) contains all variables, and the results show that the coefficient of the interaction term is still significantly negative, indicating that financial subsidies and tax incentives have inhibitory effects on the innovation efficiency of NEV enterprises in a distorted factor market.

**Table 10:** The regression results of the market distortion.

	(1)	(2)	(3)
Variables	RD	RD	RD
Subsidy	0.005** (0.002)		0.004* (0.002)
Distortation	-0.028*** (0.005)	-0.034* (0.002)	-0.026** (0.013)
Subsidy×Distortation	-0.004** (0.002)		-0.005*** (0.005)
Tax		0.098*** (0.009)	0.078*** (0.012)
Tax×Distortation		-0.095* (0.057)	-0.088** (0.044)
Size	0.001 (0.002)	0.002 (0.002)	0.002 (0.002)
Roe	0.072** (0.028)	0.066** (0.028)	0.033** (0.027)
Cash	0.005** (0.002)	0.004** (0.002)	0.001 (0.002)
Share	-0.004 (0.003)	-0.007** (0.003)	-0.009*** (0.003)
Tdr	0.004** (0.002)	0.001 (0.002)	0.002 (0.002)
Age	0.007*** (0.002)	0.006*** (0.002)	0.005** (0.002)
Labor	0.062** (0.027)	0.070** (0.028)	0.071*** (0.028)
Far	-0.001 (0.001)	0.007 (0.001)	0.012 (0.002)
TobinQ	0.015*** (0.003)	0.018*** (0.003)	0.001 (0.005)
Constant	0.031*** (0.011)	0.045** (0.022)	0.023** (0.011)
Observations	6162	6162	6162
R-squared	0.536	0.658	0.641
Region fixed effects	yes	yes	yes
Time fixed effects	yes	yes	yes

Note: Clustering robust standard errors are in parentheses, clustering at the individual enterprise level.

*, ** and *** denote coefficients significant at the 10%, 5% and 1% levels, respectively

## 5 Conclusions and suggestions

Based on the sample of Chinese listed NEV enterprises from 2010 to 2022, this paper analyzes the impact of financial subsidies and tax incentives on innovation efficiency. It investigates the different impacts under different circumstances. Also, this paper draws the following conclusions.

Both financial subsidies and tax incentives can promote NEV enterprises to increase R&D investment and output, thus enhancing innovation efficiencies. Compared to financial subsidies, tax incentives are more effective.

2. The interaction between financial subsidies and tax incentives weakens the innovation efficiency of NEV enterprises, indicating that the effect of financial subsidies and tax incentives on innovation efficiency mainly depends on their respective policies. In contrast, the combined effect of policies is weak.

3. The innovation effect of financial subsidies and tax incentives is more substantial for NEV enterprises with higher financing constraints. In addition, the ownership of enterprises also affects innovation efficiency, and the incentive effects on private enterprises’ innovation are more potent than that of SOEs.

4. Further research finds that optimizing the market-oriented environment can effectively strengthen the innovation efficiency of NEV enterprises, while the distortion of the factor market will weaken the innovation efficiency.

Our study made some contributions to the current literature.

First, this study empirically explored the impact of financial subsidies and tax incentives on innovation efficiency in the Chinese listed NEV enterprises. Previous scholars have scrutinized their linkage in the various fields(Hewitt 2010; Bøler 2015; Wu 2022; Pu 2023). The conclusions drawn regarding the effects of financial subsidies and tax incentives on innovation efficiency are also different. The empirical research in this article shows that both financial subsidies and tax incentives have improving effects on innovation efficiency, and the role of tax incentives is more significant. The result implies that the government strengthens financial subsidy support and fully leverages the role of tax preferential policies.

Second, our study focuses on the innovation efficiency of different financing constraints and ownerships. From the extant works, scholars proposed that different conditions would affect innovation efficiency(Acharya and Xu 2013). Our empirical research shows that NEV enterprises with higher financial constraints can improve innovation efficiency through financial subsidies and tax incentives. The result means that in implementing financial subsidies and tax incentives to improve the innovation efficiency of NEV enterprises, more attention should be paid to those enterprises with higher financial constraints.

Finally, this article also explores the impact of marketization and factor distortion on the innovation efficiency of NEV enterprises. Scholars have conducted meaningful research on economic transformation and innovation efficiency(Laincz 2009; Antonelli 2013).Our empirical research shows that marketization and factor distortion impact the innovation efficiency of NEV enterprises. The result requires the government to gradually improve market-oriented efficiency while reducing factor distortions to improve the innovation efficiency of NEV enterprises when implementing financial subsidies and tax incentives.

This paper proposes the following suggestions.

In policy optimization, the government should take tax incentives as the primary option and utilize the positive effects better. Compared to financial subsidies, tax incentives have the advantages of greater certainty and universality. They are more confident and have a longer-term incentive for NEV enterprises to increase innovation efficiency. It is necessary to focus on the practical implementation of the existing tax incentives to ensure that the relevant enterprises can obtain the corresponding tax incentives. Besides, the government should consider further expanding the scope of tax incentives and radiation, and strengthening the tax incentives for NEV enterprises with higher R&D investment. For example, tax incentives for small and medium-sized enterprises with higher innovative R&D expenditures should be deeply implemented and appropriately reduce the deduction standard for R&D expenses to reduce the burden of R&D expenses. In addition, the tax incentives can be strengthened to promote innovation efficiency and link the management departments of taxation, economic and information technology, science and technology to strengthen the promotion of tax incentives and reduce the threshold for enterprises to obtain tax incentives.

The government should use the financial subsidy policy as a suboptimal policy to promote the innovation efficiency of NEV enterprises. Gradual changes from direct financial subsidies to indirect subsidies and a strict audit system should be implemented and fully disclose the conditions related to financial subsidies. Before financial subsidies are implemented, the eligibility of subsidized enterprises should be fully examined. After subsidizing, it is also necessary to strengthen the follow-up evaluation and assess the subsequent impact of financial subsidies on innovation investment. To further reduce the "rent-seeking" space in the process of financial subsidies, the government should also strengthen the supervision of financial subsidies to ensure that they can effectively enhance innovation efficiency.

The government should construct a better business environment and strengthen the role of financial subsidies and tax incentives to enhance the innovation efficiency of NEV enterprises. Firstly, the government should strengthen the rule of law, strengthen the construction of an intellectual property protection system, and protect the innovation achievements of enterprises. Secondly, with the implementation of financial subsidies and tax incentives, it is necessary to clarify the incentive criteria targets, to establish an independent regulatory body. Finally, the marketization process should be accelerated, let the market play a decisive role in resource allocation, and decentralize the government to promote NEV enterprises’ innovation and development.

## References

[1] Bakhtiari S, Breunig R, Magnani L, Zhang J. Financial Constraints and Small and Medium Enterprises: A Review. Econ Rec. 2020;96: 506–523.

[2] Urbano D, Audretsch D, Aparicio S et al. Does entrepreneurial activity matter for economic growth in developing countries? The role of the institutional environment. Int Entrep Manag J. 2020;16: 1065–1099.

[3] Dimos C, Pugh G, Hisarciklilar M, Talam E, Jackson I. The relative effectiveness of R&D tax credits and R&D subsidies: A comparative meta-regression analysis. Technovation. doi: 10.1016/j.technovation.2021.102450

[4] Wangsa I, Vanany I, Siswanto N. The optimal tax incentive and subsidy to promote electric trucks in Indonesia: Insight for government and industry. Case Stud Transp Pol. doi: 10.1016/j.cstp.2023.100966

[5] Barrachina R, Moreno R. A possible mechanism for partial crowding-out of R&D subsidies in developing countries. Rev Dev Econ. 2023;6:1–26.

[6] Hewitt N, Roper S. Output Additionality of Public Support for Innovation: Evidence for Irish Manufacturing Plants, Eur Plan Stud. 2010;18:107–122

[7] Bøler E, Moxnes A, Helene K. R&D, International Sourcing, and the Joint Impact on Firm Performance. Am Econ Rev. 2015;105:3704–3739.

[8] Pu X, Zeng M, Zhang W. Corporate sustainable development driven by high-quality innovation: Does fiscal decentralization really matter?. Econ Anal Policy. 2023;78:273–289

[9] Liu S, Du J, Zhang W, Tian X, Kou G. Innovation quantity or quality? The role of political connections. Emerging Markets Review.2021;48:89–101.

[10] Gerarden Todd D, Richard G Newell, Robert N. Assessing the Energy-Efficiency Gap. J Econ Lit. 2017;55: 1486–1525.

[11] Wang X, Sun K, Xiao Z. Industrial Policy and the Rise of China’s Strategic Emerging Industries. December 30, 2022[Cited 2023 September 21].Available from: https://www.aeaweb.org/conference/2023/program/paper/SQSRZ3Bk.

[12] Xie Y, Boadu F, Tang H. Does internationalization encourage state-owned enterprises to utilize subsidies to innovate? Evidence from high-tech and automobile manufacturing industries of Chinese listed companies. Chin Manag Stud. 2022; 4: 803–829.

[13] Ovaere M, Proost S. Cost-effective reduction of fossil energy use in the European transport sector: An assessment of the Fit for 55 Package. Energy Policy. 2022; 168:113085.

[14] Wu Z, Fan X, Zhu B, Xia J, Zhang L, Wang P. Do government subsidies improve innovation investment for new energy firms: A quasi-natural experiment of China’s listed companies. Technol Forecast Soc. 2022; 175:121418. doi: 10.1016/j.techfore.2021.121418

[15] Boeing P, Eberle J, Howell A. The impact of China’s R&D subsidies on R&D investment, technological upgrading and economic growth. Technol Forecast Soc. 2022; 174: 121–142.

[16] Shi J, Sadowski, Zeng BM et al. Picking winners in strategic emerging industries using government subsidies in China: the role of market power. Humanit Soc Sci Commun. 2023; 10:394–411.

[17] Lee G Branstetter, Li GW, Ren MJ. Picking winners? Government subsidies and firm productivity in China. J Comp Econ. 2023; 5:41–63.

[18] Tong Y, Yuan Z, Chen X. Research on China’s fiscal and taxation policy of new energy vehicle industry technological innovation. Econ Res-Ekon Istraz. 2023; 36:1–28.

[19] Busom I, Corchuelo B, Martínez-Ros E. Tax incentives or subsidies for business R&D?. Small Bus Econ. 2014; 43: 571–596.

[20] Qin S, Xiong Y. Differential impact of subsidised and nonsubsidized policies on the innovation of new energy vehicle enterprises: evidence from China. Asian J Technol Inno. 2022; 31:1–24.

[21] Liu D, Chen T, Liu X, Yu Y. Do more subsidies promote greater innovation? Evidence from the Chinese electronic manufacturing industry. Econ Model. 2019; 80:441–452.

[22] Howell A. Picking winners in China: Do subsidies matter for indigenous innovation and firm productivity?. China Econ Rev. 2017; 44:154–165.

[23] Dai XY, Chapman G. R&D tax incentives and innovation: Examining the role of programme design in China. Technovation. doi: 10.1016/j.technovation.2021.102419

[24] Keizer JA, Johannes IM, Halman Michael S. From experience: applying the risk diagnosing methodology. J Prod Innovat Manag. 2002; 19:213–232.

[25] González X, Jaumandreu J, Pazó C. Barriers to Innovation and Subsidy Effectiveness. Rand J Econ. 2005; 36: 930–949

[26] Wang H, Shi J, Imran M, Gao J, Zhang Y, Wang R. The Effect of Government Subsidies on Firm R&D Investment in China: From Perspectives of Ownership and Market Power. Complexity. doi: 10.1155/2022/4905287

[27] Yang G, Liu J, Lian P, Rui M. Tax incentives, research and development manipulation, and research and development performance. Econ Res. 2017; 08:110–124

[28] Le T, Adam B. Jaffe. The impact of R&D subsidy on innovation: evidence from New Zealand firms. Econ Innov New Tech. 2016; 26:429–452.

[29] Hellmann Thomas, Thiele. Incentives and Innovation: A Multitasking Approach. American Economic Journal: Microeconomics. 2011; 3: 78–128.

[30] Acharya V, Zhang X. Financial Dependence and Innovation: The Case of Public versus Private Firms. December 2013[Cited 2023 September 21]. Available from: https://pages.stern.nyu.edu/~sternfin/vacharya/public_html/pdfs/private_Acharya_Xu.pdf.

[31] Fazzari S, Hubbard RG, Petersen B. Financing Constraints and Corporate Investment. Brookings Pap Eco Ac. 1988; 1:141–195.

[32] Song Z, Storesletten K, Zilibotti F. Growing like china. Am Econ Rev. 2011; 101: 196–233.

[33] Hadlock CJ, Pierce JR. New Evidence on Measuring Financial Constraints: Moving Beyond the KZ Index. Rev Financ Stud. 2010; 5:1909–1940.

[34] Fee CE, Hadlock CJ, Pierce J R. Investment, financing constraints, and internal capital markets: Evidence from the advertising expenditures of multinational firms. Rev Financ Stud. 2009; 6: 2361–2392.

[35] Mohammadzadeh N, Zegordi SH, Kashan AH, Nikbakhsh E. Optimal government policy-making for the electric vehicle adoption using the total cost of ownership under the budget constraint. Sustain Prod Consump. 2022; 33:477–507.

[36] Wang Z, Li X, Xue X, Liu Y. More government subsidies, more green innovation? The evidence from Chinese new energy vehicle enterprises. Renew Energ. 2022; 197:11–21.

[37] Noll B, Schmidt S, Steffen B. Analyzing the competitiveness of low-carbon drive-technologies in road-freight: A total cost of ownership analysis in Europe. Appl Energ. 2022; 306:79–118.

[38] Aloulou F, Ghannouchi I. The impact of ownership and contractual practice on the technical efficiency level of the public transport operators: An international comparison. Res Transp Bus Manag. doi: 10.1016/j.rtbm.2021.100707

[39] Yao X, Ma S, Bai Y, Jia N. When are new energy vehicle incentives effective? Empirical evidence from 88 pilot cities in China. Transport Res A-Pol. 2022; 165:207–224.

[40] Li W, Zhang X. Can the Exemption of the New Energy Vehicle Purchase Tax Policy Induce Technological Innovation of Automobile Companies?. Environ Sci Pollut Res. doi: 10.1007/s11356-023-29489-3 37656300

[41] Laincz CA. R&D subsidies in a model of growth with dynamic market structure. J Evol Econ. 2009; 19:643–673.

[42] Antonelli C, Crespi F, Scellato G. Internal and external factors in innovation persistence. Econ Innov New Tech. 2013; 3:256–280.

